# Clostridium butyricum and Bifidobacterium pseudolongum Attenuate the Development of Cardiac Fibrosis in Mice

**DOI:** 10.1128/spectrum.02524-22

**Published:** 2022-11-01

**Authors:** Jiaqi Wang, Jiahuan Chen, Linquan Li, Huanyu Zhang, Daxin Pang, Hongsheng Ouyang, Xuemin Jin, Xiaochun Tang

**Affiliations:** a College of Animal Sciences, Jilin University, Changchun, People’s Republic of China; b Chongqing Research Institute of Jilin University, Chongqing, People’s Republic of China; c State Key Laboratory for Zoonotic Diseases, Key Laboratory for Zoonosis Research of the Ministry of Education, Institute of Zoonosis, and College of Veterinary Medicine, Jilin University, Changchun China; Jilin University

**Keywords:** cardiac fibrosis, gut microbiome, butyric acid, mixed probiotics

## Abstract

Cardiac fibrosis is an integral aspect of every form of cardiovascular diseases, which is one of the leading causes of death worldwide. It is urgent to explore new effective drugs and treatments. In this paper, transverse aortic constriction (TAC)-induced cardiac fibrosis was significantly alleviated by a cocktail of antibiotics to clear the intestinal flora, indicating that the gut microbiota was associated with the disease process of cardiac fibrosis. We transplanted feces from sham-operated and TAC-treated mice to mice treated with a cocktail of antibiotics. We found that TAC-treated gut microbiota dysbiosis cannot cause cardiac fibrosis on its own. Interestingly, healthy fecal microbiota transplantation could alleviate cardiac fibrosis, indicating that targeted probiotics and related metabolite intervention may restore a normal microenvironment for the treatment or prevention of cardiac fibrosis. We used 16S rRNA sequencing of fecal samples and discovered that butyric acid-producing bacteria and Bifidobacterium pseudolongum were the dominant bacteria in the group with the lowest degree of cardiac fibrosis. Moreover, we demonstrated that sodium butyrate prevented the development of cardiac fibrosis. The effect of Clostridium butyricum (butyric acid-producing bacteria) was better than that of B. pseudolongum on cardiac fibrosis. Surprisingly, the cocktail of two probiotics had a stronger ability than a single probiotic. In conclusion, therapies targeting the gut microbiota and metabolites such as probiotics present new strategies for treating cardiovascular disease.

**IMPORTANCE** Cardiac fibrosis is a basic process in cardiac remodeling. It is related to almost all types of cardiovascular diseases (CVD) and has become an important global health problem. Basic research and a number of clinical studies have shown that myocardial fibrosis can be prevented and reversed to a certain extent. It is urgent to explore new effective drugs and treatments. We indicated a causal relationship between cardiac fibrosis and gut microbiota. Gut microbiota dysbiosis cannot cause cardiac fibrosis on its own. Interestingly, healthy fecal microbiota transplantation could alleviate cardiac fibrosis. According to our findings, the combined use of butyric acid-producing bacteria and B. pseudolongum can help prevent cardiac fibrosis. Therapies targeting the gut microbiota and metabolites, such as probiotics, represent new strategies for treating cardiovascular disease.

## INTRODUCTION

Cardiac fibrosis is a basic process in cardiac remodeling. It is related to almost all types of cardiovascular diseases (CVD) and has become an important global health problem (1). Basic research and a number of clinical studies have shown that myocardial fibrosis can be prevented and reversed to a certain extent (2–7). Myofibroblast activation and increased extracellular matrix (ECM) deposition in the interstitial space are hallmarks of cardiac fibrosis. Interstitial fibroblasts create collagen for the creation of the ECM network, which provides structural support for the heart while also allowing systolic force transfer (8–10). Accordingly, drugs such as spironolactone and ginsenoside Rg2 inhibited ECM and collagen deposition (11–15). However, the effect of these drugs on the recovery of cardiac function index associated with fibrosis is limited. The side effects associated with the long-term use of existing drugs also limit their application in the treatment of fibrosis (16). Therefore, it is necessary to explore new therapies and effective treatments.

The gut microbiota inhabiting the gastrointestinal tract promotes health by decreasing susceptibility to infection and enhancing resistance to a range of diseases (17). Some studies contributed to the establishment of the “gut hypothesis of heart failure,” which proposes that dysregulation of the gut microbiota could contribute to adverse outcomes in patients with heart disease (18–21). New options for treating CVD include therapies that target the gut microbiota and metabolites such as probiotics and short-chain fatty acids (SCFAs) (22, 23). Probiotics have been demonstrated to reduce myocardial infarct size (24), atherosclerotic plaque area (25), and the incidence rates of postinfarction myocardial hypertrophy and heart failure (26). There is increasing evidence that the intestinal microbiota may play a role in the biological process of triggering and maintaining essential hypertension, which is associated with CVDs (27). The ratio of *Firmicutes*/*Bacteroides* increased and acetate- and butyrate-producing bacteria were depleted in patients with hypertension (28). SCFAs, one of the major metabolites in the gut, are the end product of gut dietary fiber metabolism and influence a variety of physiological processes (29). SCFA has a wide range of functions, from immune and metabolic regulation to reducing atherosclerosis (30). Emerging evidence supports that the intestinal microbiota is involved in cardiac fibrosis (31, 32). It is unclear whether the recovery of intestinal function and microbiota composition may have a beneficial impact on cardiac fibrosis. More research is needed to improve the formulation and efficacy so that probiotic therapy can become a viable clinical approach to fibrosis-related diseases.

In this study, we explored the relationship between the flora and cardiac fibrosis to find a strategy for targeting intestinal microorganisms to prevent and treat cardiac fibrosis. We performed an experiment to remove the flora and perform fecal transplantation in a transverse aortic constriction (TAC) mouse model. A specifically tailored multistrain probiotic targeting the gut microbiota represents a new strategy to treat CVD.

## RESULTS

### The gut microbiota was involved in cardiac fibrosis.

**FIG 1:**
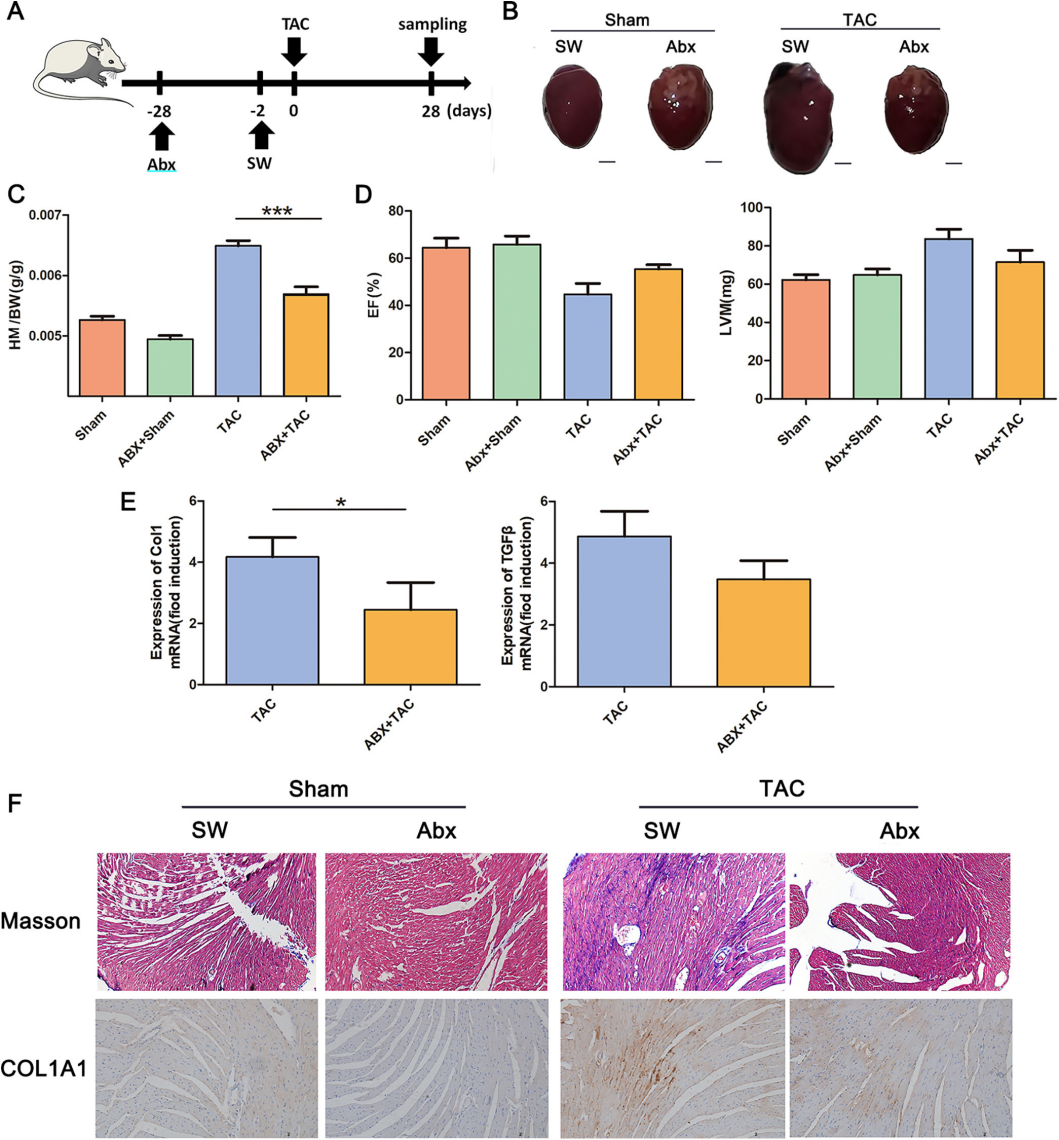
Loss of the gut microbiota alleviated cardiac fibrosis. (A) Experiment-specific operation time flow chart. (B) Whole hearts for each group (*n* = 7). (C) Heart mass-to-body weight ratio of mice in each group. Representative images are shown. (D) Ejection fraction and left ventricular weight obtained by cardiac ultrasound in each group of mice. (E) qPCR analysis of Col1 and TGF-β expression in the heart tissue of mice in each group. The levels of Col1 and TGF-β in tissues from the sham group were assigned a value of 1.0. Different mRNA expression levels among the TAC, ABX+sham, ABX+TAC and sham groups were compared by one-way ANOVA. *, *P* < 0.05. (F) Masson staining and COL1A1 immunohistochemistry results of sham-operated (sham) or TAC(TAC) mouse hearts after microbiota clearance (ABX+sham, ABX+TAC). Magnification, ×200. Representative images are shown.

Four weeks after TAC surgery in mice, our results demonstrated successful cardiac fibrosis modeling. As expected, TAC induced left ventricular hypertrophy (Fig. 1B) and a significant increase in the heart mass-to-body weight ratio (HM/BW) (Fig. 1C). TAC resulted in a decrease in ejection fraction (EF) and increased left ventricular weight (LVM) (Fig. 1D). Compared to sham operation, TAC significantly increased type I collagen and transforming growth factor β (TGF-β) mRNA expression (Fig. 1E). In order to evaluate the degree of interstitial and perivascular fibrosis, the cross section of the left ventricle was stained by Masson trichrome staining. Compared with the control group, we observed an increase in interstitial collagen deposition caused by TAC. This result was also confirmed by the immunohistochemical staining for collagen type I alpha 1 chain (COL1A1) (Fig. 1F). However, the above-described symptoms were significantly alleviated by the cocktail of antibiotic treatments applied to clear the intestinal flora.

**FIG 2:**
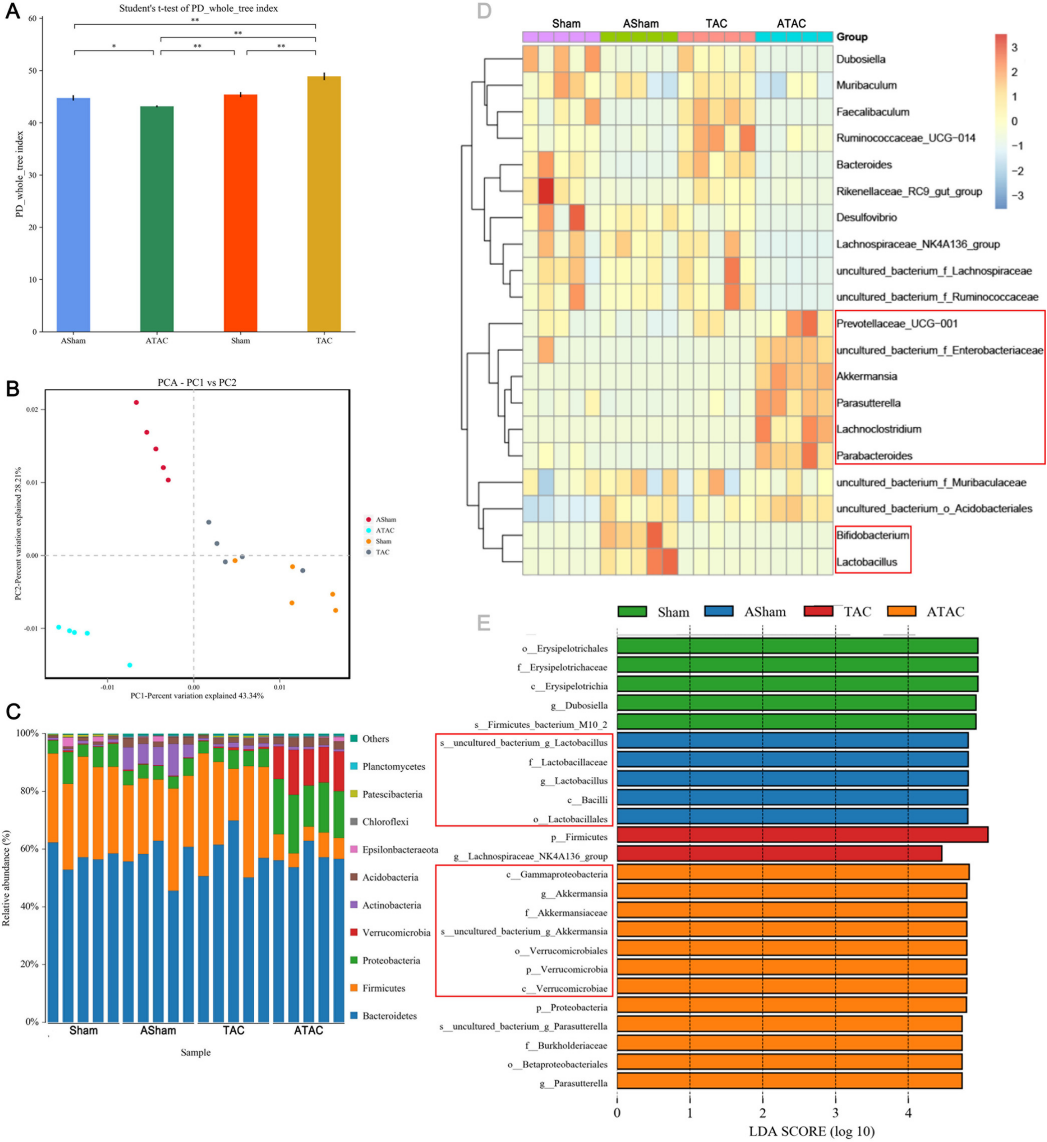
Characterization of the gut microbiota in sham-operated and TAC-operated mice with or without microbiota clearance. The feces of five mice from each group were randomly selected, and the microbiota composition of the small intestine contents was analyzed by 16S rRNA gene sequencing. (A) Shannon index (α diversity). (B) Principal-coordinate analysis (PCoA) plot (β diversity). (C) Composition of abundant bacterial phyla. (D) Linear discriminant analysis (LDA) effect size showing the most significantly differentially abundant taxa enriched in the microbiota from the sham (sham), ABX+sham (ASham), TAC (TAC), and ABX+TAC (ATAC) groups. (E) Heatmap of the abundances of bacteria at the genus level.

We investigated the effects of cardiac fibrosis (CF) on the gut microbiome using 16S rRNA gene sequencing. TAC significantly increased the α diversity (Shannon index). The α diversity was significantly reduced following clearance and then TAC (Fig. 2A). We observed a clear separation between the α diversities after surgeries with and without bacterial clearance (Fig. 2B). The antibiotic cocktail (ABX)+TAC group showed a significantly decreased relative abundance of *Firmicutes* and increased abundances of *Verrucomicrobia* and *Proteobacteria* compared to the TAC group. The ABX+sham group showed significantly increased abundance of *Actinobacteria* compared to the sham group (Fig. 2C). At the genus level, *Enterobacteriaceae*, *Akkermansia*, and *Parasutterella* were enriched in the ABX+TAC group. *Bifidobacterium* and *Lactobacillus* were enriched in the ABX+sham group (Fig. 2D). The dominant bacterium *Akkermansia* (belonging to *Verrucomicrobia*) was significantly expanded in the ABX+TAC group, suggesting that this genus in the intestinal flora may play a role in alleviating cardiac fibrosis. Moreover, *Firmicutes* were enriched in the gut bacterial community in the TAC group, indicating that the bacteria may promote cardiac fibrosis (Fig. 2E). This finding suggests that these bacteria may have a role in alleviating cardiac fibrosis.

### TAC-induced gut microbiota dysbiosis does not result in cardiac fibrosis.

**FIG 3:**
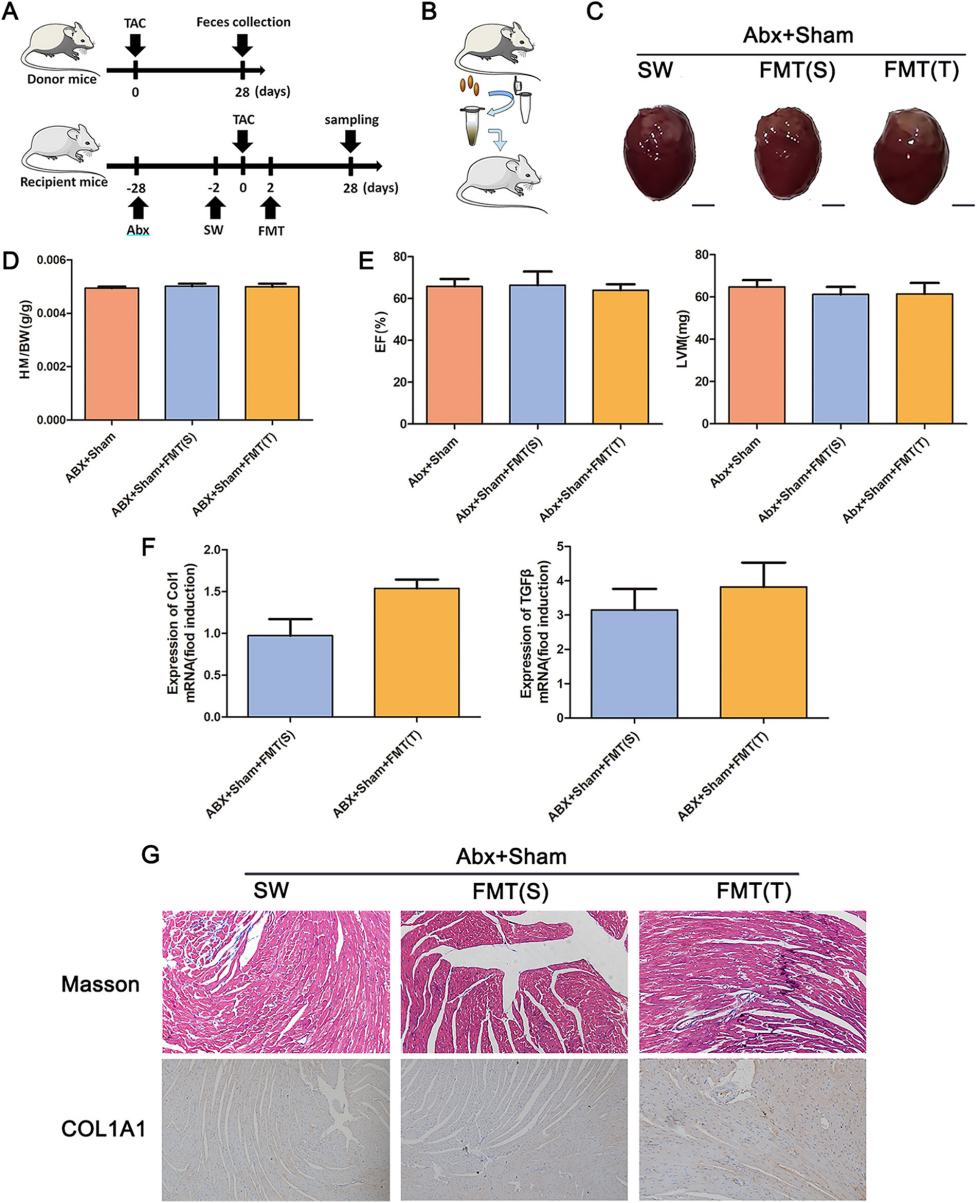
TAC-induced gut microbiota dysbiosis does not result in cardiac fibrosis. (A) Experiment-specific operation time flow chart. (B) Schematic diagram of fecal microbiota transplantation (FMT). (C) Whole hearts for each group (*n* = 7). (D) Heart mass-to-body weight ratio of mice in each group. Representative images are shown. (E) Ejection fraction and left ventricular weight obtained by cardiac ultrasound in each group of mice. (F) qPCR analysis of Col1 and TGF-β expression in the heart tissue of mice in each group. The levels of Col1 and TGF-β in tissues from the ABX+sham group were assigned a value of 1.0. Different mRNA expression levels among the ABX+sham+FMT(S), ABX+sham+FMT(T), and ABX+sham groups were compared by one-way ANOVA. *, *P* < 0.05. (G) Masson staining and COL1A1 immunohistochemistry results of the fecal source of FMT received by depleted mice after sham (ABX+sham), either sham [ABX+sham+FMT(S)] or TAC mice [ABX+sham+FMT(T)]. Magnification, ×200. Representative images are shown.

To further demonstrate the causal relationship between the gut microbiota and cardiac fibrosis, we transplanted feces from sham-operated and TAC-treated mice after 4 weeks into mice treated with a cocktail of antibiotics (Fig. 3A). There were no significant differences in left ventricular hypertrophy, the HM/BW, the EF, or the LVM in the mice, indicating that the gut microbiota alone cannot cause cardiac fibrosis. The mRNA expression levels of type I collagen and TGF-β, Masson’s trichrome staining, and COL1A1 immunohistochemistry results were different, but not significantly (Fig. 3). The α diversity of the gut microbiota showed that the microbiota diversity in mice transplanted with feces from TAC-treated mice was higher than that in mice transplanted with feces from sham-operated mice. The β diversity results showed that the bacterial diversity of mice transplanted with TAC mouse feces was significantly different from that of mice transplanted with sham mouse feces (Fig. 3H).

**FIG 4:**
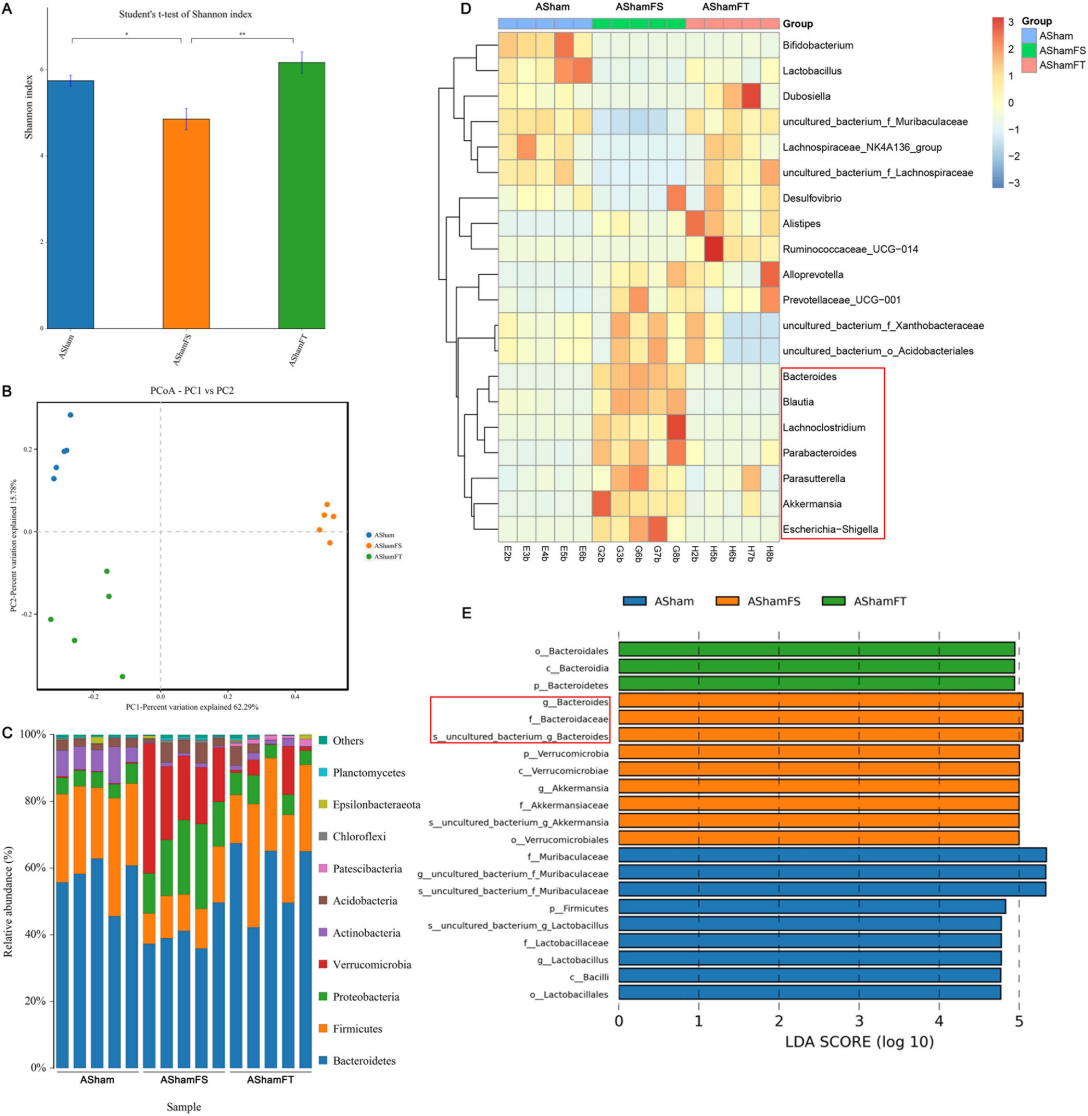
Characterization of the gut microbiota in mice cleared of their microbiota and undergoing sham surgery with or without FMT. The feces of five mice from each group were randomly selected, and the microbiota composition of the small intestine contents was analyzed by 16S rRNA gene sequencing. (A) Shannon index (α diversity). (B) Principal-coordinate analysis (PCoA) plot (β diversity). (C) Composition of abundant bacterial phyla. (D) Linear discriminant analysis (LDA) effect size showing the most significantly differentially abundant taxa enriched in the microbiota from the ABX+sham (ASham), ABX+sham+FMT(S) (AShamFS), and ABX+sham+FMT(T) (AShamFT) groups. (E) Heatmap of the abundances of bacteria at the genus level.

We investigated the effects of fecal microbiota transplantation (FMT) on the gut microbiome using 16S rRNA gene sequencing. As expected, the feces of sham-transplanted mice showed significantly decreased α diversity, and the feces of TAC-transplanted mice showed significantly increased α diversity (Fig. 4A). We observed a clear separation among the groups transplanted with flora from different mice (Fig. 4B). The ABX+sham+FMT(S) group showed significantly decreased *Actinobacteria* and *Firmicutes* abundances and increased *Verrucomicrobia* and *Proteobacteria* abundances compared to those of the ABX+sham group. The ABX+sham+FMT(T) group showed significantly decreased abundances of *Verrucomicrobia* and *Acidobacteria* compared to that of the ABX+sham group (Fig. 4C). These changes were confirmed by heatmap (Fig. 4D) and between-group difference histogram analyses (Fig. 4E).

### Healthy fecal microbiota transplantation interventions attenuated cardiac fibrosis.

**FIG 5:**
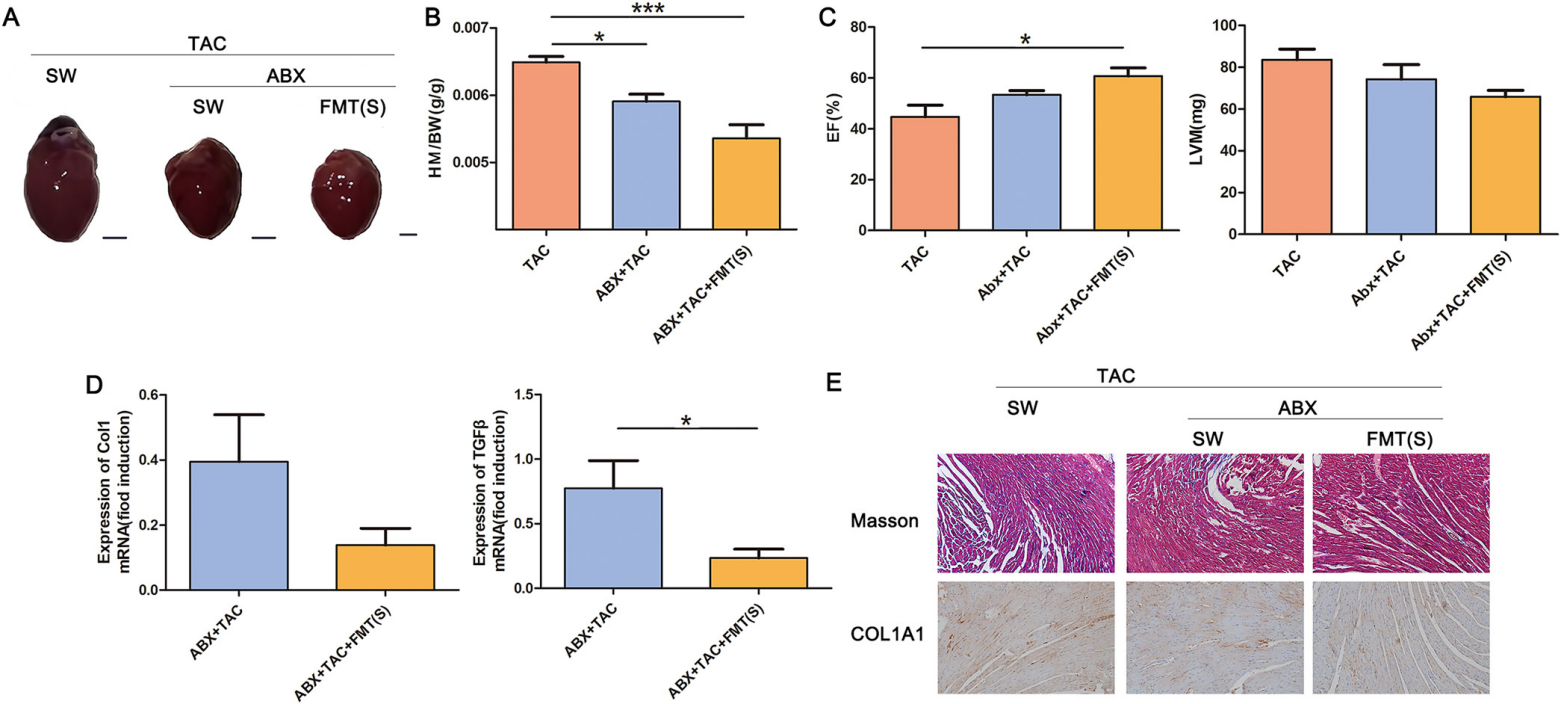
FMT ameliorated cardiac fibrosis. (A) Whole hearts for each group (*n* = 7). (B) Heart mass-to-body weight ratio of mice in each group. Representative images are shown. (C) Ejection fraction and left ventricular weight obtained by cardiac ultrasound in each group of mice. (D) qPCR analysis of Col1 and TGF-β expression in the heart tissue of mice in each group. The levels of Col1 and TGF-β in tissues from the TAC group were assigned a value of 1.0. Different mRNA expression levels among the ABX+TAC, and ABX+TAC+FMT(S) and TAC groups were compared by one-way ANOVA. *, *P* < 0.05. (E) Masson staining and COL1A1 immunohistochemistry results of the mice that underwent TAC surgery (TAC) after bacterial clearance (ABX+TAC) and received FMT in sham mice [ABX+TAC+FMT(S)]. Magnification, ×200. Representative images are shown.

Although the gut microbiota cannot directly cause cardiac fibrosis, we next asked whether the microbiota can serve as a regulatory target to alleviate the progression of cardiac fibrosis. We transplanted feces from mice 4 weeks after sham surgery into mice with TAC after gut microbiota clearance. We found that the increase in left ventricular hypertrophy and the HM/BW induced by TAC was significantly attenuated by FMT (Fig. 5A, B). Compared to the TAC group, FMT improved the EF and increased LVM (Fig. 5C), while it reduced mRNA expression of type I collagen and TGF-β (Fig. 5D). Masson’s trichrome staining and COL1A1 immunohistochemistry results indicated that FMT prevented the excessive collagen deposition promoted by TAC (Fig. 5E). Bacterial flora difference analysis showed that there were significant differences in the bacterial flora of the three groups of mice (Fig. 5F). We hypothesized that the gut microbiota can serve as a therapeutic target for cardiac fibrosis.

**FIG 6:**
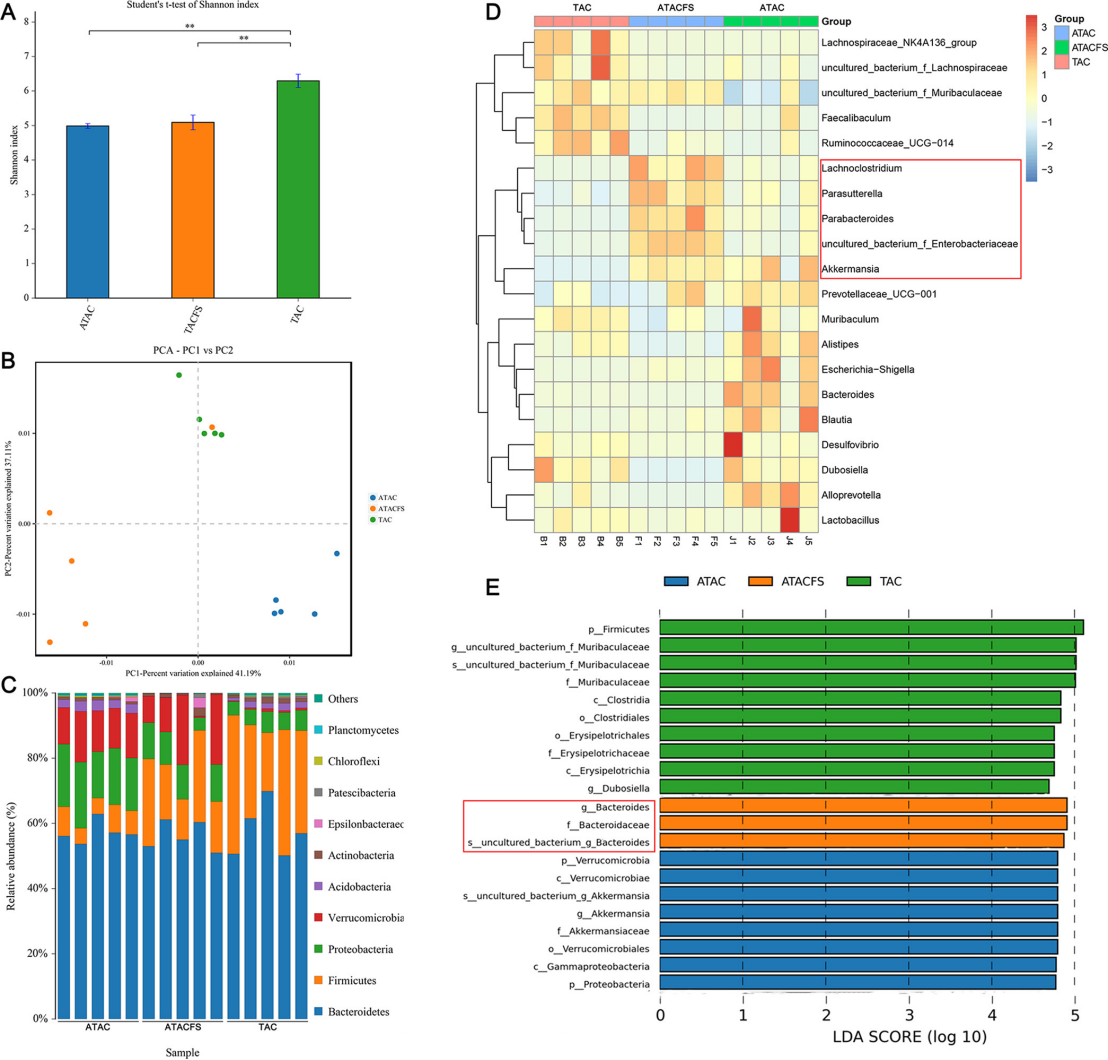
Characterization of the gut microbiota in mice cleared of the microbiota followed by FMT after TAC surgery or sham surgery. The feces of five mice from each group were randomly selected, and the microbiota composition of the small intestine contents was analyzed by 16S rRNA gene sequencing. (A) Shannon index (α diversity). (B) Principal-coordinate analysis (PCoA) plot (β diversity). (C) Composition of abundant bacterial phyla. (D) Linear discriminant analysis (LDA) effect size showing the most significantly differentially abundant taxa enriched in microbiota from the TAC (TAC), ABX+TAC (ATAC), and ABX+TAC+FMT(S) (ATACFS) groups. (E) Heatmap of the abundances of bacteria at the genus level.

We investigated the effects of different degrees of mitigation of cardiac fibrosis on the gut microbiome using 16S rRNA gene sequencing. Consistent with previous results, the ABX+TAC group and ABX+TAC+FMT(S) group had significantly decreased α diversity (Fig. 6A). We observed a clear separation between mice that underwent surgeries with and without bacterial clearance (Fig. 6B). The ABX+TAC group and ABX+TAC+FMT(S) group showed a significantly decreased relative abundance of *Firmicutes* and increased relative abundances of *Verrucomicrobia* and *Proteobacteria* compared to those of the TAC group (Fig. 6C). At the genus level, *Lachnoclostridium*, *Parabacteroides*, and *Enterobacteriaceae* were enriched in the ABX+TAC group. *Bacteroides* and Escherichia*-Shigella* were enriched in the ABX+TAC+FMT(S) group. Interestingly, *Akkermansia* and *Prevotellaceae*_UCG-001 (belonging to the *Bacteroidetes*) were enriched in both groups (Fig. 6D). We found from the between-group difference histogram that the main difference in the bacterial community in the ABX+TAC group was the *Verrucomicrobia* abundance, and the main difference in the bacterial community in the ABX+TAC+FMT(S) group was the *Bacteroides* abundance (Fig. 6E). Both types of bacteria produce SCFAs.

### Sodium butyrate prevented the development of cardiac fibrosis.

**FIG 7:**
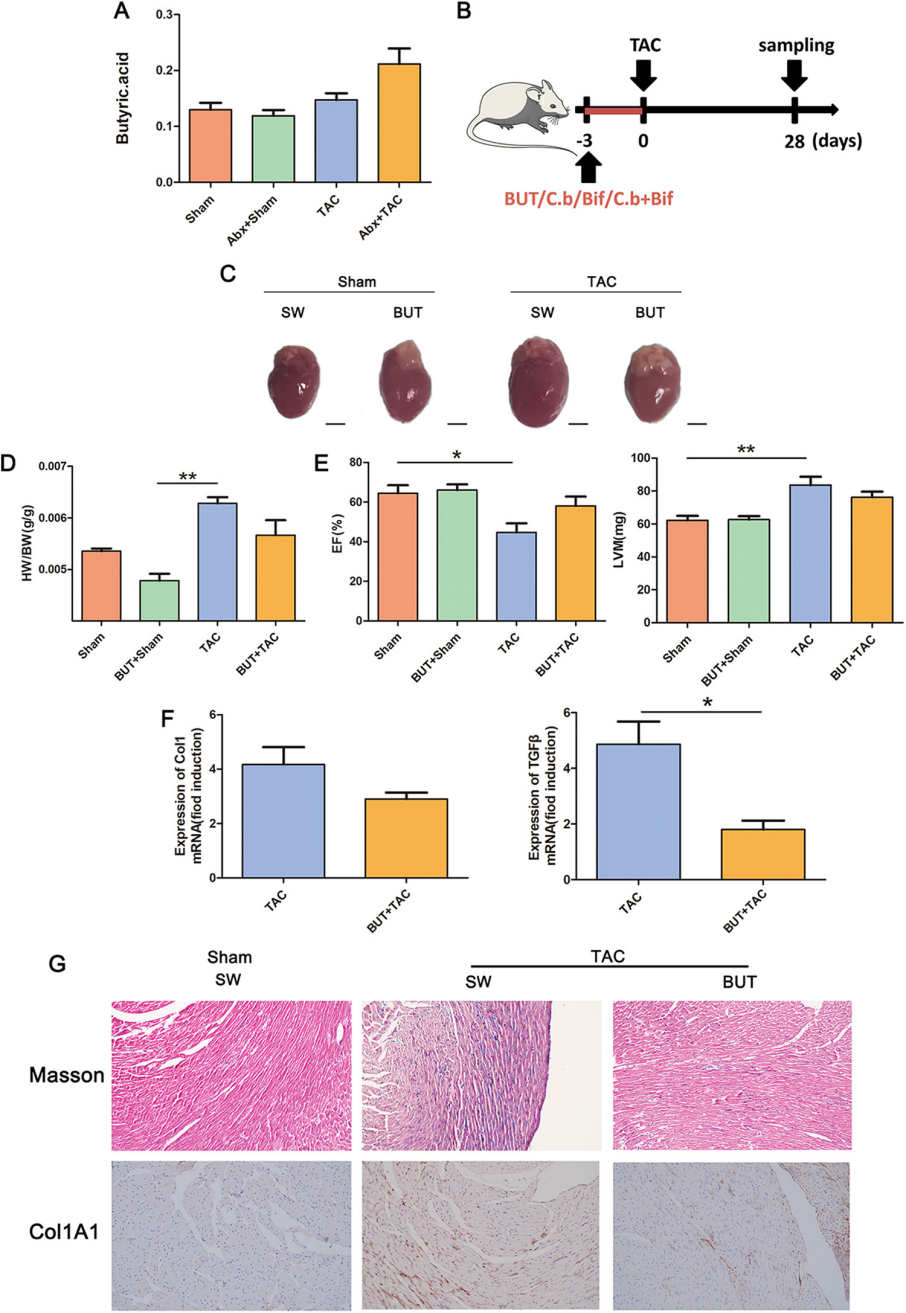
Sodium butyrate ameliorated cardiac fibrosis. (A) Serum levels of butyric acid in mice in each group. (B) Experiment-specific operation time flow chart. (C) Whole hearts for each group (*n* = 7). (D) Heart mass-to-body weight ratio of mice in each group. Representative images are shown. (E) Ejection fraction and left ventricular weight obtained by cardiac ultrasound in each group of mice. (F) qPCR analysis of Col1 and TGF-β expression in the heart tissue of mice in each group. The levels of Col1 and TGF-β in tissues from the sham group were assigned a value of 1.0. Different mRNA expression levels among the TAC, BUT+TAC, and sham groups were compared by one-way ANOVA. *, *P* < 0.05. (G) Masson staining and COL1A1 immunohistochemistry results of sham-operated (sham) or TAC mouse (TAC) hearts were obtained after intraperitoneal injection of sodium butyrate (BUT+sham, BUT+TAC) Magnification, ×200. Representative images are shown.

To explore how the microbiota relieves cardiac fibrosis, we analyzed the microbiota of different groups and found that there were more SCFA-producing bacteria in the microbiota in the sham operation group and after microbiota transplantation (Fig. 7). Therefore, we determined the levels of SCFAs and found that butyric acid is more abundant in the serum of mice with remission of cardiac fibrosis (Fig. 7A; see Fig. S1 in the supplemental material). Therefore, we continually intraperitoneally injected butyric acid into mice for 3 days before TAC model establishment (Fig. 7B). Cardiac fibrosis was significantly alleviated in the butyric acid-treated mice (Fig. 7C to G), indicating that sodium butyrate played a role in preventing cardiac fibrosis.

### A cocktail of probiotics attenuated development in two models of cardiac fibrosis.

**FIG 8:**
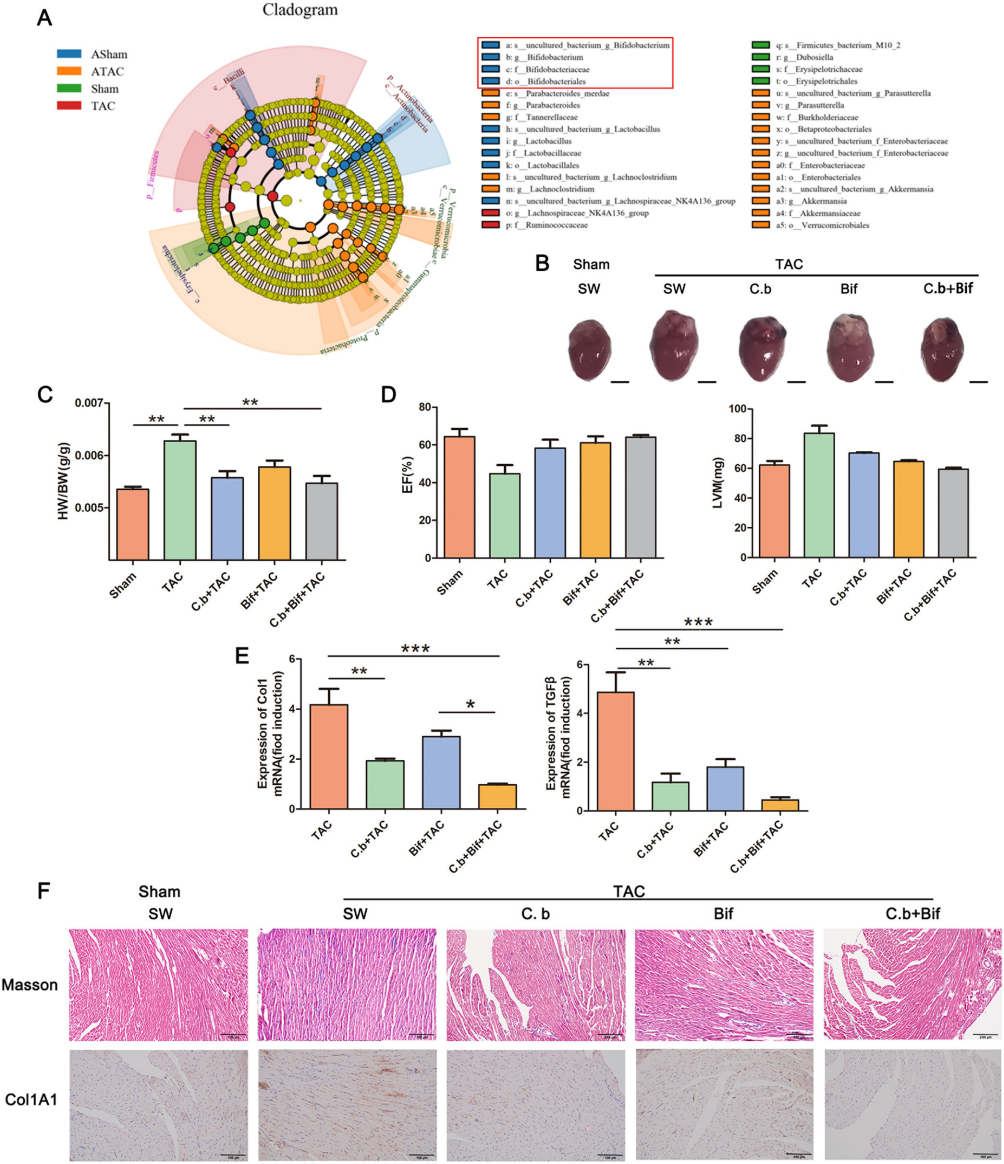
Combined use of *C. butyricum* and B. pseudolongum effectively improves cardiac fibrosis. (A) Clade map of LEfSe analysis of mouse fecal microbes in each group. (B) Whole hearts for each group (*n* = 7). (C) Heart mass-to-body weight ratio of mice in each group. Representative images are shown. (D) Ejection fraction and left ventricular weight obtained by cardiac ultrasound in each group of mice. (E) qPCR analysis of Col1 and TGF-β expression in the heart tissue of mice in each group. The levels of Col1 and TGF-β in tissues from the sham group were assigned a value of 1.0. Different mRNA expression levels among the TAC, C.b+TAC, Bif+TAC, C.b+Bif+TAC, and sham groups were compared by one-way ANOVA. *, *P* < 0.05. (F) Masson staining and COL1A1 immunohistochemistry results of mouse hearts of mice subjected to oral gavage with *C. butyricum* (C.b+TAC), B. pseudolongum (Bif+TAC), or *C. butyricum* and B. pseudolongum (C.b+Bif+TAC) before TAC surgery (TAC) or sham operation (Sham). Magnification, ×200. Representative images are shown.

To improve the application prospect of using butyric acid to prevent cardiac fibrosis, we used the butyric acid-producing bacterium *C. butyricum* to prevent cardiac fibrosis. The linear discriminant analysis effect size (LEfSe) algorithm demonstrated that *Bifidobacterium* was the dominant bacterium in the group with the lowest degree of cardiac fibrosis (Fig. 8A). Therefore, we evaluated the efficacy of a cocktail of *C, butyricum* and B. pseudolongum on cardiac fibrosis. We showed that both probiotics can alleviate cardiac fibrosis and that the effect of *C. butyricum* was better than that of B. pseudolongum. Surprisingly, the combined use of both probiotics had a stronger ability than the application of a single probiotic (Fig. 8B to F).

In addition, helminth infection induced left ventricular hypertrophy (Fig. S2A) and a significant increase in the heart mass-to-body weight ratio (HM/BW) (Fig. S2B). Helminth infection resulted in a decrease in EF and increased LVM (Fig. S2C), and Trichinella spiralis significantly increased type I collagen and TGF-β mRNA expression (Fig. S2D). Compared with the control group, we observed an increase in interstitial collagen deposition caused by helminth infection. This result was also confirmed by the immunohistochemical staining for COL1A1 (Fig. S2E). Interestingly, we found that the cocktail of probiotics also alleviated helminth-induced cardiac fibrosis in mice.

## DISCUSSION

Cardiac fibrosis is a basic process of cardiac remodeling. It is related to almost all types of cardiovascular diseases and has become a major global health problem (1). Cardiac fibrosis not only accelerates disease progression, but it also plays a key role in treatment failure in clinical cardiovascular trials. Although several commonly used medications have been shown to be clinically beneficial in the treatment of fibrosis (11, 33–36), these drugs have limited effect on the recovery of cardiac function index related to fibrosis. In addition, they are not suitable for long-term use (16). Although cardiac fibrosis is common, there are still no effective drugs to provide sufficient clinical intervention for cardiac fibrosis (37). New drugs and effective treatments are needed.

Cardiac fibrosis is characterized by excess extracellular matrix (ECM) deposition in cardiac tissue. Fibrotic tissue is harder and less pliant, leading to subsequent cardiac insufficiency and heart failure (38). Cardiac injury causes the release of bioactive TGF-β from dormant stores via mechanisms involving proteases, integrins, and specialized ECM proteins (39). The production of transforming growth factor β (TGF-β) isoforms is upregulated and activated in myocardial diseases. These factors play an important role in cardiac repair and remodeling by regulating the phenotype and function of cardiomyocytes, fibroblasts, immune cells, and vascular cells (39). Based on heart size, HM/BW, echocardiography, Masson staining, and COL1 and TGF-β expression, we found that after 4 weeks of TAC modeling, significant cardiac fibrosis occurred in the mouse heart, and the degree of cardiac fibrosis was alleviated after removing the flora, which was consistent with previous research results (31). Because the intestinal flora is linked to cardiac fibrosis (32), we performed a fecal bacterial transplantation experiment to investigate the relationship between the two. The flora from animals with cardiac fibrosis could not cause cardiac fibrosis. This finding differs from the results for hypertension (40) and other diseases (41–43), all of which can be driven by the gut microbiota. We anticipated that the formation of cardiac fibrosis may require prolonged stimulation of bacterial flora disturbance or coinduction of other conditions. We transplanted the feces of sham-operated mice into the model mice after removing the flora. We found that the cardiac fibrosis of mice induced by TAC was significantly ameliorated after FMT, suggesting that intestinal flora might be used as a therapeutic target for cardiac fibrosis.

The intestinal flora regulates the host primarily through flora-produced metabolites (44). According to a LEfSe analysis of an evolutionary branching diagram, the main functional bacteria of the cardiac fibrosis remission group were *Bacteroides* and *Akkermansia* in the *Verrucomicrobia*, which are bacteria that produce and regulate SCFAs. Therefore, we detected the level of SCFAs in mouse serum. The results showed that butyric acid content increased in the group with remission of cardiac fibrosis, which is consistent with the increase in butyric acid levels accompanied by the increase in *Bacteroides* or *Akkermansia* abundance in many reports (45–48). The results showed that after administration of sodium butyrate, indicators such as heart size were relieved. The expression level of TGF-β was decreased after butyrate administration, consistent with the results of some previous articles (49–52). However, some articles have suggested that butyrate has a synergistic effect with TGF-β and that butyrate administration increases the level of TGF-β expression (53–55). Interestingly, many studies have shown that butyric acid can alleviate myocardial infarction (MI), glucose dysmetabolism, and uric acid-associated cardiac tissue damage (56, 57). Our study shows that butyric acid can alleviate cardiac fibrosis caused by TAC. It has been shown to exert beneficial functions in the maintenance of systemic regulation of host immune response by increasing anti-inflammatory factors, downregulating autoimmunity-related factors, and developing regulatory T (Treg) cells via G-protein-coupled receptors (GPCRs) (e.g., GPR41, GPR43, and GPR109A) (58–61).

Probiotics are the most common application means for targeting the intestinal flora. We used the butyric acid-producing bacterium *C. butyricum* for the prevention and treatment of cardiac fibrosis to better apply the regulatory effect of butyric acid on cardiac fibrosis. In addition, *Bifidobacterium* was the dominant bacterium in the group with the lowest degree of cardiac fibrosis. The intervention of combined live *C. butyricum* and *Bifidobacterium* is safe and well tolerated (62). In our study, we used *C. butyricum* and B. pseudolongum to prevent TAC-induced cardiac fibrosis. The findings showed that B. pseudolongum alone can alleviate the symptoms of cardiac fibrosis, but not significantly, whereas a cocktail of probiotics has the best preventive effect and the lowest degree of cardiac fibrosis, which could be attributed to the combination of the two bacteria enhancing the efficiency of lactic acid use for the production of butyric acid (63). Furthermore, this cocktail of probiotics has also shown efficacy in helminth-induced cardiac fibrosis, which is infective cardiac fibrosis.

These results suggest that the intestinal flora is involved in cardiac fibrosis but that it cannot induce cardiac fibrosis alone. Transplanting a healthy intestinal flora can alleviate cardiac fibrosis. The intestinal metabolite butyric acid is the key to preventing cardiac fibrosis. According to our findings, the combined use of butyric acid-producing bacteria and B. pseudolongum can help prevent cardiac fibrosis. Therapies targeting the gut microbiota and metabolites, such as probiotics, represent new strategies for treating cardiovascular disease.

## MATERIALS AND METHODS

### Ethics statement.

All animal studies and the breeding process were carried out in accordance with guidelines approved by the Animal Welfare and Research Ethics Committee of Jilin University. All animals were maintained on standard rodent chow with water supplied *ad libitum* and with a 12/12 h light/dark cycle during the experimental period (64). The protocol was approved by the Institutional Animal Care and Use Committee of Jilin University.

### Animal study design.

Male C57BL/6J mice (8 weeks old) were randomized into two groups (*n* = 7) to receive either TAC or sham surgery (sham). Two groups (*n* = 7) of mice were randomly selected to receive a cocktail of antibiotics (ampicillin, 1 g/L; metronidazole, 1 g/L; vancomycin, 0.5 g/L; neomycin, 0.5 g/L) in their drinking water for 28 days to clear the intestinal flora and were fed with sterile water for 2 days before undergoing TAC surgery (antibiotic cocktail [ABX]+TAC) or sham surgery (ABX+sham). Two groups (*n* = 7) of mice were randomly selected to remove the intestinal flora, fed with sterile water for 2 days, and then subjected to sham operation and transplanted with feces 4 weeks after sham operation [ABX+sham+FMT(S)] and TAC [ABX+sham+FMT(T)] operation. A group (*n* = 7) of mice was randomly selected to clear the intestinal flora, fed sterile water for 2 days before TAC surgery, and transplanted with feces 4 weeks after sham surgery [ABX+TAC+FMT(S)]. All the above-described groups underwent stool collection, cardiac ultrasonography, and heart and body weighing, and serum and heart samples were collected 4 weeks after surgery.

According to the results of the above-described experiments, several groups of animal experiments were set up. The 8-week-old male C57BL/6J mice were randomized into two groups (*n* = 7) to receive either TAC or sham surgery (sham). Two groups (*n* = 7) of mice were randomly selected to undergo continual intraperitoneal injection of sodium butyrate (100 mg/kg 0.9% saline) for 3 days and then underwent sham surgery (BUT+sham) or TAC surgery (BUT+TAC). Six groups (*n* = 7) of mice were randomly selected for continual intragastric administration of *C. butyricum*, B. pseudolongum, or a combination of the two (1 × 10^7^ CFU/200 μL 0.9% saline) for 3 days and then underwent sham surgery (C.b+sham/Bif+sham/C.b+Bif+sham) or TAC surgery (C.b+TAC/Bif+TAC/C.b+Bif+TAC). All the above-described groups underwent cardiac ultrasonography and heart and body weighing, and serum and heart samples were collected 4 weeks after the operation.

The helminth Trichinella spiralis isolate (ISS534) was obtained from a naturally infected domestic pig in Henan Province, China. Briefly, Wistar rats were orally infected with 3,000 infective larvae, and *T. spiralis* muscle larvae (ML) were recovered at 35 days postinfection (dpi) (65). Then, 8-week-old male C57BL/6J mice were randomized into 3 groups with 6 in each group. The mice in the control group were uninfected. For the mice in the helminth-infected group, gavage was performed with 300 *T. spiralis*. The mice in treatment group were administered a combination of *C. butyricum* and B. pseudolongum (1 × 10^7^ CFU/200 μL 0.9% saline) for 3 days at 14 dpi. All the mice were sacrificed at 28 dpi, and their organs were taken for further analysis.

### Transverse aortic constriction.

TAC surgery was performed as previously described (66). Briefly, the animals were anesthetized with a mixture of ketamine (2.5%) and xylazine (0.2%), at doses of 150 mg/kg and 12 mg/kg, respectively, diluted in sterile saline and administered by intraperitoneal injection. After induction of anesthesia, the mice were placed in the supine position under a dissecting stereoscope and subjected to a tracheotomy. After that, the animals underwent endotracheal intubation and were artificially ventilated with a constant volume of 0.5 mL and 110–120 breaths/min respiratory rate (687 series respirator, Harvard Apparatus). The respiratory rhythm and thoracic mobility were used to verify the success of intubation. A left-sided parasternal thoracotomy was performed between the second and third left intercostal spaces, and the transverse aorta was isolated after thymus separation. The aorta was tied with a 7-0 cardiovascular silk suture (FST) between the origins of the innominate artery and left common carotid artery against a 27-gauge needle positioned perpendicular to the vessel, which served as a template to determine the degree of constriction. After that, a double knot was made, the needle was removed, and the chest wall was closed with a 4-0 silk suture. The corresponding sham-operated (sham) mice underwent the same procedure without ligation of the aorta. After TAC surgery, all animals were conditioned for 30 min in medical oxygen (2 mL/min) for recovery. The mice were euthanized 28 days after TAC or sham surgery using CO_2_ (67).

### Fecal microbiota transplantation (FMT).

The process of fecal microbiota transplantation was performed as previously described (68, 69). The microbiota donor mice were treated with sham or TAC surgery, and 4 weeks later, feces were collected daily. Feces from each group were pooled, and 200 mg was resuspended in 2 mL of sterile saline. The mixture was vigorously vortexed for 10 s before centrifugation at 800 × *g* for 3 min. The supernatant was collected and used as transplant material. Meanwhile, 8-week-old male C57BL/6 J mice were randomly divided into three groups (7 mice/group). After acclimation for 1 week, mice were treated with the antibiotic cocktail (ABX: ampicillin, 1 g/L; metronidazole, 1 g/L; vancomycin, 0.5 g/L; neomycin, 0.5 g/L) (70) in drinking water for 4 weeks to create pseudo-microbiota-depleted mice. The sham-operated and TAC-treated microbiota-depleted mice were subjected to oral gavage with the fecal suspension (150 μL) once per day for 1 week and every 2 days for 3 weeks (71).

### Echocardiography.

Transthoracic echocardiography was performed to measure cardiac function (72). Then, 4 weeks after TAC, mice were subjected to transthoracic echocardiography using a high-frequency MyLab Six ultrasound system equipped with a variable frequency 8- to 16-MHz probe (MyLab, Jilin, China). The animals were anesthetized with isoflurane (5%) in an inhalation chamber and maintained anesthetized with a dose of 1.25% isoflurane. The parasternal short-axis view was obtained at the level of the papillary muscles. Left ventricle (LV) internal dimensions at diastole/systole (LVIDd/LVIDs) and LV anterior/posterior wall thickness (LVAW/LVPW) were measured and used to calculate the ejection fraction (EF). Echocardiographic acquisition and echocardiographic data analysis were performed by an observer (Ziyang Yu) blinded to treatment.

### Tissue collection.

Mice were weighed 4 weeks after surgery. Fecal specimens were snap-frozen in liquid nitrogen and stored at −80°C until further testing. At the time of euthanasia, the heart was immediately removed and weighed. Apical tissue was removed and fixed in a 4% formaldehyde solution, and serum was also collected and stored at −80°C until further testing.

### Microbial analysis in feces.

Genomic DNA amplification and sequencing were conducted. PCR amplification was performed as described previously (73, 74). Briefly, microbial DNA was extracted from the fecal contents of the mice. Then DNA was stored at −80°C until further processing. The V3-V4 region of the bacterial 16S rRNA gene was amplified with the common primer pair (forward primer, 5′- ACTCCTACGGGAGGCAGCA-3′; reverse primer, 5′-GGACTACHVGGGTWTCTAAT-3′) combined with adapter sequences and barcode sequences.

The bioinformatics analysis of this study was performed with the aid of BMK Cloud (Biomarker Technologies Co., Ltd., Beijing, China). We processed the raw data with Trimmomatic (v0.33) to trim low-quality reads. Cutadapt was used to discard forward and reverse primers. The overlapping paired-end reads were merged to a single sequence using FLASH. The UCHIME algorithm (v8.1) was used to detect and remove chimera sequences to obtain the clean tags. Sequences with similarity of ≥97% were clustered into the same operational taxonomic unit (OTU) using USEARCH (v10.0), (75) and the OTUs with relative abundance of <0.005% were filtered. The DADA2 (76) method in QIIME 2 (77) (v2020.06) was applied to de-noise sequences, generating amplicon sequence variant (ASV). The conservative threshold for OTU filtration is 0.005%. Taxonomy was assigned to all OTUs by searching against the Silva (release 132, http://www.arb-silva.de) databases using the RDP classifier within QIIME 2. The α diversity and β diversity were calculated and displayed using QIIME and R software, respectively. The relative abundances were analyzed. Furthermore, we employed linear discriminant analysis (LDA) effect size (LEfSe). Based on the Kyoto Encyclopedia of Genes and Genomes (KEGG) functional pathways, the functional composition was predicted for each sample using Phylogenetic Investigation of Communities by Reconstruction of Unobserved States 2 (PICRUSt2) (78). Statistical analyses were conducted with STAMP, and functional differences in orthologs among groups were assessed by a one-way analysis of variance (ANOVA) followed by Tukey-Kramer multiple comparisons (74, 79, 80).

### Short-chain fatty acids.

Serum samples were collected for quantification of SCFAs using an Agilent 7890A gas chromatograph (Agilent Technologies, USA) at Suzhou Bionovegene Co., Ltd., Jiangsu, China (71).

### Immunohistochemistry.

The tissue sections were baked in a 60°C incubator for 1 h, after which they were quickly transferred to xylene I and allowed to rest for 15 min, xylene II for 10 min, absolute ethanol for 5 min, and 95% and 75% ethanol, each for 5 min. Then the tissues were immersed in running water for 5 to 10 min, heated in 95°C antigen retrieval solution for 30 to 45 min, and slowly cooled down to room temperature. The sections were then transferred into phosphate-buffered saline (PBS) for 5 to 10 min. A freshly prepared 3% H_2_O_2_ methanol solution was added to the slide (at room temperature for 10 min). Next, the sections were washed with PBS (2 min × 3 times), blocked with goat serum (PH0424; Phygene Biotechnology, Fuzhou, China), and incubated at room temperature for 20 min. The diluted primary antibody, rabbit anti-mouse COL1A1 (Boster, Wuhan, China), was added to the sections, and the slides were placed in a wet box and incubated at 4°C overnight. Rabbit serum was used as a negative control (NC) at the same time. After washing with PBS (2 min × 5 times), a biotin-labeled goat anti-rabbit secondary antibody (Beyotime Biotechnology, Shanghai, China) was added to the sections and incubated for 10 min, followed by two PBS washes. Then, a solution of horseradish peroxidase-conjugated streptavidin (21140; Pierce, Rockford, IL) was added to the sections for 10 min of incubation, followed by washing with PBS (2 min × 5 times). Next, 3,3′-diaminobenzidine (DAB; ADI-950-211-0200; Assay Designs, Guangzhou, China) was applied for color development for 1 to 2 min. Hematoxylin staining was employed to counterstain the nucleus for 1 to 1.5 min, and the slides were then washed slowly with running water. Afterward, the sections were differentiated by 0.1% hydrochloric acid and alcohol, colorized to blue, dehydrated, and cleared. The sections were allowed to rest in ethanol (75%, 95%, 100%, separately), xylene I, and xylene II for 1, 1, 1, 2, and 2 min, respectively. After being dried with xylene, the sections were sealed with neutral gum and photographed under a microscope (81, 82).

### Masson staining.

Paraffin sections were cut into 4-μm sections and stained with Masson’s trichrome to analyze the collagen present in heart samples. The liver tissue sections were baked at 60°C for 90 min; immersed in xylene (3 min × 3 times), absolute ethanol (3 min × 2 times), and 95% and 75% ethanol (each for 3 min), and flushed with running water. Then, the sections were stained with hematoxylin for 10 min, flushed with running water, differentiated with hydrochloric acid for several seconds, flushed with running water again, and colorized to blue with ammonia for several minutes. Next, Ponceau-acid fuchsin, 12-molybdophosphoric acid solution, and green staining solution were successively used to stain the sections for 4 to 20 s, 2 to 4 min, and 2 to 5 min, respectively. After being washed with water and baked in an oven, the sections were sealed with neutral gum and observed and photographed under a microscope. In each section, five fields of view were chosen for observation under the microscope (×200) (81, 83, 84).

### Tissue gene expression.

We examined the mRNA expression levels of TNF-α and Cola1 in heart mRNA using real-time PCR. The primers for TGF-β1-F (279 bp) were as follows: 5′-ACCTGCAAGACCATCGACAT-3′ and 5′-GGTTTTCTCATAGATGGCGT-3′. The primers for collagen I-F (310 bp) were as follows: 5′-TCCAAAGGAGAGCGGTAA-3′ and 5′-GACCAGGACACA-3′. The relative mRNA expression levels of the target genes were normalized to those of the indicated housekeeping gene (GAPDH, 108 bp) (5′-TGAAGGGGTCGTTGATGG-3′ and 5′-AAATGGTGAAGGTCGGTGTG-3′) and were quantified using the comparative threshold cycle *C_T_* method and the formula 2^–ΔΔ^*^CT^*.

### Statistical analysis.

The GraphPad Prism (v5) package was used for statistical analysis and graphing. Two-tailed independent sample *t* tests (with Welch correction in the case of different variances) and analysis of variance (ANOVA with Tukey adjustment for multiple comparisons) were used to compare the data between 2 and 3 groups, respectively. Differences were considered significant at *P* < 0.05.

### Data availability.

Microbiome sequencing data have been deposited at the National Center for Biotechnology Information Sequence Read Archive (PRJNA723732).
