# Supplementary material for: Clostridium butyricum and Bifidobacterium pseudolongum Attenuate the Development of Cardiac Fibrosis in Mice
Source: Microbiol Spectr. 2022 Nov 1;10(6):e02524-22. doi: 10.1128/spectrum.02524-22 (PMC9769846; doi:10.1128/spectrum.02524-22)
Supplement: Supplemental file 1 — Fig. S1 and S2. Download spectrum.02524-22-s0001.pdf, PDF file, 0.7 MB [file spectrum.02524-22-s0001.pdf]

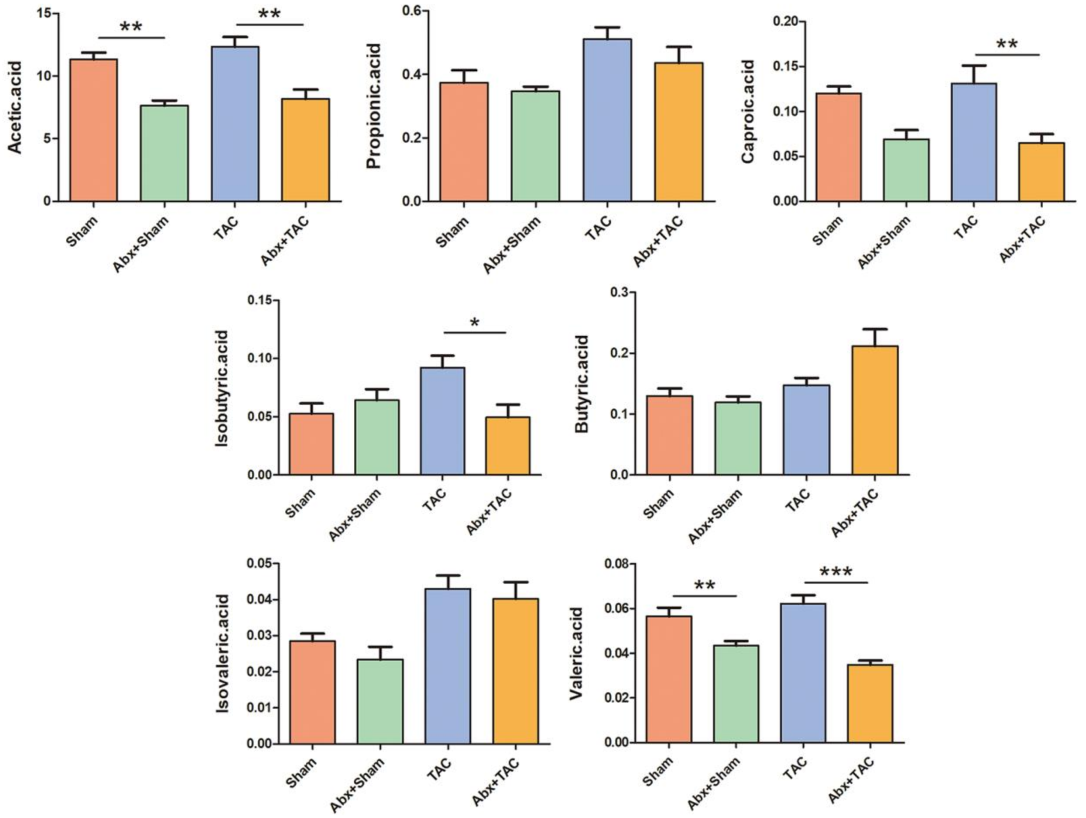

Fig. S1 The levels of SCFA in serum of mice in each group. Bars show the mean ±SD (n=5) of three independent experiments. Different SCFA expression levels among the TAC, ABX+Sham, ABX+TAC and Sham groups were compared by one-way ANOVA. *, P < 0.05.

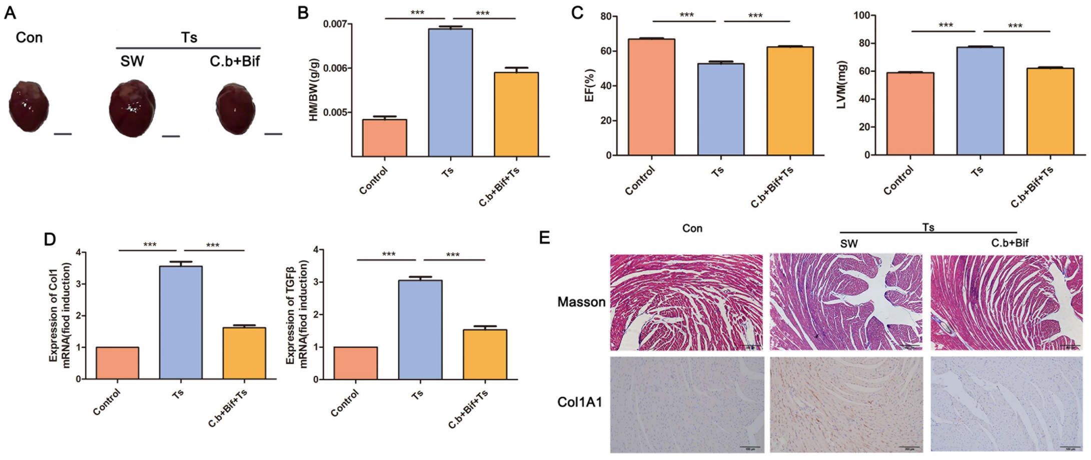

Fig. S2 Combined use of *C butyricum* and *B pseudolongum* effectively improves cardiac fibrosis induced by helminth infection. (A) Whole heart for each group (n=6). (B) Heart mass-to-body weight ratio of mice in each group. Representative

images are shown. (C) Ejection fraction and left ventricular weight obtained by cardiac ultrasound in each group of mice. (D) qPCR analysis of Col1 and TGF-β expression in the heart tissue of mice in each group. The levels of Col1 and TGF-β in tissues from the Control group were assigned a value of 1.0. Different mRNA expression levels among the Ts, C.b+Bif+Ts and Control groups were compared by one-way ANOVA. *, $P < 0.05$. (F) Masson staining and COL1A1 immunohistochemistry results of mouse hearts of mice gavaged with *C. butyricum* and *.B pseudolongum* (C.b+Bif+Ts) after *T. spiralis* (Ts) and Control (Control). Magnification, 200×. Representative images are shown.
